# Tetherin Restricts Herpes Simplex Virus 1 and Is Antagonized by Glycoprotein M

**DOI:** 10.1128/JVI.02250-13

**Published:** 2013-12

**Authors:** Caroline Blondeau, Annegret Pelchen-Matthews, Petra Mlcochova, Mark Marsh, Richard S. B. Milne, Greg J. Towers

**Affiliations:** University College London, Medical Research Council Centre for Medical Molecular Virology, Division of Infection and Immunity, University College London, London, United Kingdoma; MRC Laboratory for Molecular Cell Biology, University College London, London, United Kingdomb

## Abstract

Tetherin is a broadly active antiviral effector that works by tethering nascent enveloped virions to a host cell membrane, thus preventing their release. In this study, we demonstrate that herpes simplex virus 1 (HSV-1) is targeted by tetherin. We identify the viral envelope glycoprotein M (gM) as having moderate anti-tetherin activity. We show that gM but not gB or gD efficiently removes tetherin from the plasma membrane and can functionally substitute for the human immunodeficiency virus type 1 (HIV-1) Vpu protein, the prototypic viral tetherin antagonist, in rescuing HIV-1 release from tetherin-expressing cells. Our data emphasize that tetherin is a broadly active antiviral effector and contribute to the emerging hypothesis that viruses must suppress or evade an array of host cell countermeasures in order to establish a productive infection.

## INTRODUCTION

Mammalian cells encode restriction factors that provide the host with protection against virus replication. In order to establish a productive infection, a virus must evade or suppress a repertoire of restriction factors directed against it by the host. Restriction has been studied most extensively in the context of retrovirus infections, where numerous factors and the corresponding viral antagonists have been well characterized (1). While some restriction factors, such as TRIM5α, appear to be specific for particular classes of virus, in this case retroviruses, others, such as tetherin, are broadly active against unrelated viruses. Tetherin (also known as BST-2, CD317, or HM1.24) was identified as the cellular factor responsible for suppression of Vpu-negative human immunodeficiency virus type 1 (HIV-1) (2, 3), but subsequent work has shown that it is effective against a variety of enveloped viruses (2, 4–8) that use distinct mechanisms to antagonize its restrictive effects (9–12). Tetherin is a type 2 integral membrane protein with a C-terminal GPI anchor. The antiviral activity of tetherin stems from this unusual double membrane-linked topology that allows the formation of a protein tether between the host membrane and the budding viral envelope, preventing release of nascent virions (13).

Herpesviruses, a large family of enveloped DNA viruses, are ancient pathogens thought to have coevolved with their hosts for many generations (14). As such, they might be expected to possess countermeasures to a variety of restriction factors and thus to provide a good experimental model system for studies of this aspect of the virus host interaction. To date, two members of this virus family, Kaposi's sarcoma-associated herpesvirus (KSHV) and human cytomegalovirus (HCMV), have been shown to interact with tetherin (15–17). Surprisingly, the mode of interaction differs for these two viruses, with tetherin acting as a restriction factor for KSHV but as an entry cofactor for HCMV. In this study, we investigated the effect of tetherin on another human herpesvirus, herpes simplex virus 1 (HSV-1). We show that tetherin restricts the HSV-1 replication cycle by suppressing virus release, and we identify the viral envelope glycoprotein M (gM) as a countermeasure contributing to antagonism of tetherin restriction.

## MATERIALS AND METHODS

### Cell lines, plasmids, and viruses.

HT1080 cells expressing internally hemagglutinin (HA)-tagged human tetherin (at amino acid 154) or empty vector (LHCX) are nonclonal drug-selected populations and have been described (16), as has the tetherin expression vector pCR3.1/hu-Tetherin-HA (18). The HSV-1 gM (UL10) gene was PCR amplified from HSV-1 17+-infected-cell DNA and inserted into pCDNA3. The HSV-1 gB (UL27) and gD (US6) plasmids (pSR175 and pSC390) were gifts from Roselyn Eisenberg and Gary Cohen (University of Pennsylvania) (19, 20). Plasmids expressing Vpu, in pCDNA3 (for HIV-1 release assay) or pIRESeGFP (for flow cytometry), were described previously (21). Wild-type (WT) HSV-1 SC16 and HSV-1 KOS K26GFP, encoding a VP26-green fluorescent protein (GFP) fusion protein (22) were gifts from Gillian Elliott (Imperial College London). HSV-1 with a deletion of UL10 (ΔgM) and its revertant (RgM) were gifts from Helena Browne (University of Cambridge), and their construction has been described (23).

### HSV-1 replication assay.

HT1080 cells (3 × 10^5^ cells/well, 6-well plates) were chilled to 4°C and then incubated with HSV-1 for 1 h. Plates were then refed and transferred to 37°C for a further hour. The medium was then removed and replaced with acid-citrate buffer (500 μl, pH 3.0) to inactivate extracellular virus, followed by the addition of fresh medium. Infected-cell culture supernatants were recovered at various times postinfection and centrifuged to remove cellular debris, and virus titers determined by plaque assay on Vero cells. For cell-associated virus titers, cells were lysed by 3 freeze-thaw cycles into an equal volume of medium, cleared by centrifugation, and titrated as described above. The HSV-1 proteins ICP4 and VP5 were detected in infected-cell lysates by immunoblotting using specific antibodies (Santa Cruz). As a loading control we detected β-actin (Abcam) on stripped blots.

### RNA interference.

We used lentiviral vectors encoding tetherin-specific hairpins (shRNA1, 5′-GGAGUUCUGGUGUUCCUGAUUAUUUCGAUGAUCAGGAGCACCAGAAUUCC-3′; shRNA2, 5′-GUGGGAAUCGUGGAUAAGAAGUAUUCGUACUUCUUGUCCGCGAUUCUCAC-3′; underlining indicates tetherin-targeted sequence) or a GFP hairpin (24) as a control. Depletion was examined by immunoblotting or by quantitative PCR on cDNA (see below). Cells were infected with HSV-1 96 h post-shRNA transduction as described above.

### Quantification of tetherin and HSV-1 by TaqMan PCR.

Encapsidated HSV-1 genomes were quantified by extracting total DNA from DNase I-treated supernatants or infected-cell lysates as described previously (16). DNA was subjected to quantitative TaqMan PCR (Q-PCR) for HSV-1 UL27 as described previously (25). Absolute copy number was determined by reference to a standard curve, plotted using serial dilutions of a cloned UL27 amplicon with a detection limit of 10 UL27 copies/15 μl of supernatant. Copy numbers were normalized to extracted DNA carrier concentration (supernatants) or to quantities of extracted DNA (cells). Total mRNA was extracted from transduced HeLa cells or from HT1080 cells expressing HA-tagged tetherin and infected with HSV-1 SC16 or not infected, and cDNA was synthesized for use as the template in TaqMan Q-PCRs for tetherin and GAPDH. Tetherin primers were as follows: forward, 5′-ACCTGCAACCACACTGTGATG-3′; reverse, 5′-CAAGCTCCTCCACTTTCTTTTGTC-3′; tetherin probe, 5′-FAM-CCCTAATGGCTTCCCTGGATGCAGA-TAMRA-3′. Absolute copy number was determined with reference to a standard curve derived using a tetherin-encoding plasmid. Q-PCR for GAPDH was performed as described previously (16).

### Flow cytometry.

For tetherin cell surface staining, HEK293T cells in 6-well plates were transfected (Fugene-6; Roche) with pCR3.1/hu-Tetherin-HA and 250 ng, 500 ng, or 1,000 ng of plasmids expressing gM, gD, or gB (21). A pIRES2eGFP plasmid coding for Vpu was used as a control. At 48 h posttransfection, cell surface tetherin expression was examined on unfixed live cells with an anti-HA monoclonal antibody (Covance) and analyzed by flow cytometry. The mean fluorescence intensity of tetherin staining was measured as described previously (7). HSV-1 glycoproteins were detected by immunoblotting with a rabbit anti-gM (23), a rabbit anti-gD R8 (26), or a goat anti-gB (Santa Cruz) antibody after membrane stripping. As a loading control, we detected β-actin, GAPDH (Abcam), or transferrin receptor (Invitrogen).

### HIV-1 release assay.

To prepare vesicular stomatitis virus G glycoprotein (VSV-G)-pseudotyped HIV-1 particles, 10^6^ HEK293T cells were cotransfected with the Gag-Pol expression vector p8.91 (300 ng), pMDG encoding VSV-G (300 ng), and HIV-1 vector encoding YFP (450 ng) (27). Tetherin construct (100 ng) was cotransfected along with either 250 ng, 500 ng, or 1,000 ng of HSV-1 gM, gB, or gD or HIV-1 Vpu plasmid. DNA dose was equalized with the empty vector pcDNA3 (Invitrogen). After 48 h, cell-associated p55 Gag and p24 capsid and p24 capsid in the supernatant were detected by immunoblotting as described previously (27). The intensity of p55 bands in cell lysates and p24 bands in virions was analyzed with Image Studio 3.1.4 software (LI-COR), and ratios of p55 to p24 were calculated with signal intensity percentages relative to values obtained in the absence of tetherin.

### Microscopy.

For electron microscopy, 1 × 10^5^ HT1080 cells expressing HA-tagged tetherin or control cells seeded on coverslips were infected with 2 × 10^5^ (experiment 1) or 1 × 10^5^ (experiment 2) PFU (determined on Vero cells) of HSV-1 K26GFP (HT1080 cells are an order of magnitude less permissive than Vero cells to HSV-1, and therefore multiplicities for these experiments can be estimated to be 0.2 and 0.1 PFU/cell, respectively). After 16 h, the cells were fixed for 45 min in 2% paraformaldehyde (PFA)–2% glutaraldehyde in 0.1 M sodium cacodylate buffer (pH 7.4), postfixed for 1 h on ice in 1% OsO_4_–1.5% K_3_[Fe(CN)_6_], treated with 1.5% tannic acid (TAAB Laboratories), dehydrated, and embedded in Epon 812 (TAAB). Ultrathin sections (70 nm) were cut *en face* on a Leica EM UC7 ultramicrotome, placed on Formvar-coated slot grids, and stained with lead citrate. Sections were examined with a Tecnai G2 Spirit transmission EM (FEI), and digital images were recorded with a Morada 11 MegaPixel transmission electron microscopy (TEM) camera (Olympus Soft Imaging Solutions) and ANALYSIS software. Images were adjusted for brightness and contrast, and figures were assembled with Photoshop CS. To determine the numbers of cell surface HSV-1 particles, at least 50 consecutive/adjacent cell profiles in the section were inspected for each sample. Circular profiles of HSV-1 particles, measuring between 80 and 180 nm in diameter and with the morphologies indicated in Fig. 3, were counted.

For confocal microscopy, HEK293T cells were plated on poly-l-lysine-coated coverslips in 24-well plates and transfected (Fugene-6; Roche) with plasmids encoding gM, gB, or gD and/or HA-tagged tetherin. Between 20 and 48 h later, cells were fixed (4% PFA), permeabilized (0.1% Triton X-100), and stained using anti-HA (Covance), rabbit anti-gM, rabbit anti-gD R8, goat anti-gB, or sheep anti-TGN46 (Serotec) antibodies and secondary antibodies linked to Alexa-488, -594, or -633 (Molecular Probes) or rhodamine (Pierce). Cells were observed using a Leica TCS SPE, DM2500, confocal microscope (Leica Microsystems). Images were adjusted for brightness and contrast with Adobe Photoshop software 10.0.

## RESULTS

### Tetherin restricts HSV-1 particle release.

**Fig 1:**
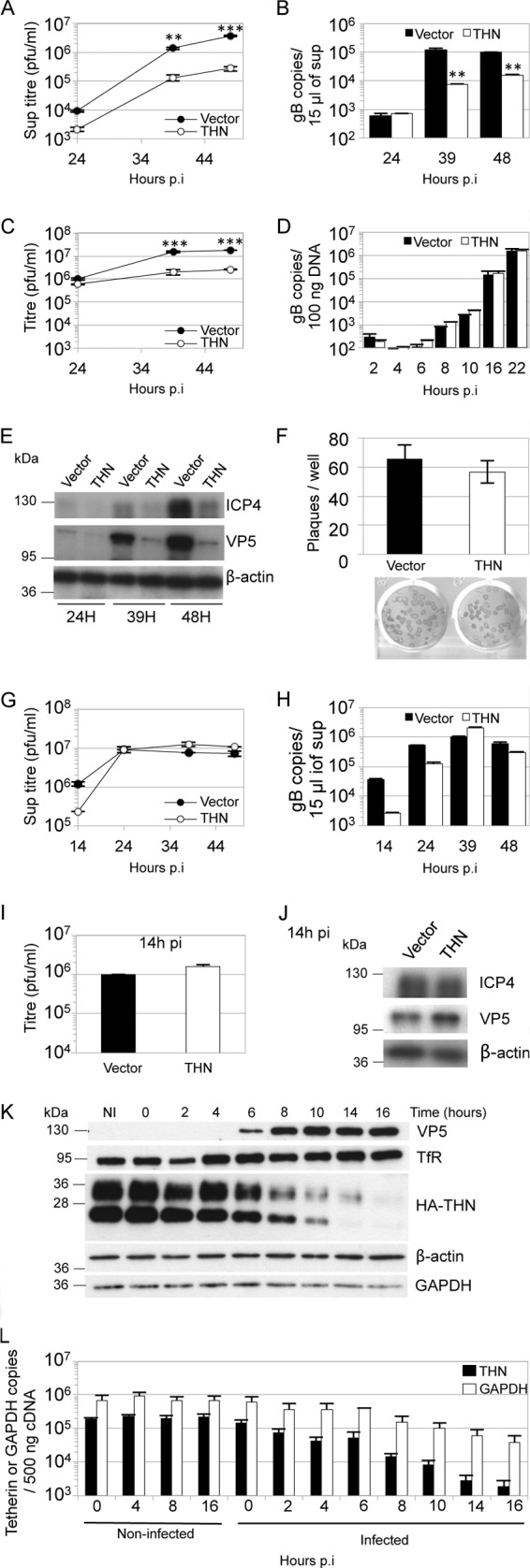
Human tetherin restricts HSV-1 particle release. HT1080 cells expressing HA-tagged tetherin (THN) or empty vector (Vector) were infected with HSV-1 SC16 at 0.01 (A to E) or 3 PFU/cell (G to J). Supernatants were harvested at the indicated times and titrated for HSV-1 infectivity by plaque assay (A and G) or subjected to DNase I treatment followed by Q-PCR for the gB gene UL27 (B and H). At the same time, cells were harvested, and freeze/thawed 3 times before titration for HSV-1 infectivity on Vero cells (C and I), extracted DNA was subjected to Q-PCR for the gB gene UL27 (D), or lysates were analyzed by immunoblotting (E and J). Statistical significance, as a *P* value, was determined by 2-way analysis of variance (ANOVA) (**, *P* < 0.01; ***, *P* < 0.001). Tetherin had no effect on DNA replication up to 22 hpi (D) (2-way ANOVA; *P* > 0.05). After high-MOI infection (3 PFU/cell), tetherin had no effect on HSV-1 supernatant titers (G) (2-way ANOVA; *P* > 0.05), or cell-associated virus titers (I) (*t* test; *P* > 0.05). (F) HT1080 cells expressing HA-tagged tetherin or empty vector were infected with HSV-1 SC16 and washed with acid citrate buffer 1 h later, before the addition of overlay medium for plaque assays. Plaques were counted 48 h later after crystal violet staining or immune-alkaline phosphatase staining (for the plaques shown). Results are means and standard errors of the means (SEM) and are from 3 independent experiments. (K, L) HT1080 cells expressing HA-tagged tetherin or empty vector were infected with HSV-1 SC16 (2 PFU/cell), and cell lysates were prepared at the indicated times and analyzed by immunoblotting to detect VP5 expression and HA tag (tetherin), transferrin receptor (TfR), β-actin, and GAPDH after membrane stripping (K), or tetherin and GAPDH mRNA levels were assessed in noninfected and infected cells by Q-PCR. Under these conditions, tetherin mRNA was not detectable in the empty vector HT1080 cells. HSV-1 infection decreased tetherin mRNA copy number (L) (2-way ANOVA; *P* < 0.01). Results are means and standard deviations (SD) and are representative of at least 2 separate experiments, except when specified.

To seek evidence for restriction of HSV-1, we expressed HA-tagged human tetherin in HT1080 cells. These cells were chosen because they naturally express very low levels of tetherin and are highly permissive for HSV-1 (28). As a control, we used HT1080 cells transduced with empty vector. We hypothesized that tetherin overexpression might saturate any anti-tetherin activities mediated by the virus and reveal tetherin sensitivity. We infected both HT1080 cell lines with HSV-1 SC16 at a low multiplicity (0.01 PFU/cell), aiming to study the effect of tetherin in a multicycle infection by measuring the titer of virus released at various times by plaque assay on Vero cells. Tetherin expression consistently reduced the levels of infectious virus released, leading to a 14-fold reduction at 48 h postinfection (hpi), compared to controls (Fig. 1A). Quantitative PCR (Q-PCR) detection of DNase I-resistant (i.e., encapsidated) HSV-1 DNA in the infected-cell culture supernatants demonstrated a comparable decrease in signal, supporting the notion that tetherin suppresses virion release from infected cells (Fig. 1B). Tetherin expression did not affect titers of cell-associated virus (Fig. 1C) or levels of cell-associated viral DNA (Fig. 1D) at early time points up to 24 hpi. At later times postinfection, titers of cell-associated virus from tetherin-expressing cells were 7- to 8-fold lower than those of controls (Fig. 1C), and consistent with this, levels of viral proteins detected by Western blotting were also reduced (Fig. 1E). We assume that at these later time points, we see the cumulative effect of tetherin's inhibition of viral release and the consequent reduction in number of newly infected cells in subsequent rounds of infection. Importantly, in a plaque assay there was no difference between tetherin-expressing and control HT1080 cells in the number, or size, of plaques obtained from a given virus dose, suggesting that tetherin had no impact on HSV-1 entry, or direct cell-to-cell spread (Fig. 1F). This is consistent with the specificity of tetherin for virus release over cell-to-cell spread, as has been described for tetherin restriction of the lentiviruses HIV-1 and feline immunodeficiency virus (FIV) (29, 30).

We next tested whether tetherin expression could suppress high-multiplicity infection. Tetherin-expressing and control HT1080 cells were infected with HSV-1 (input multiplicity, 3 PFU/cell), and the virus yield was measured at various times by plaque assay as before (Fig. 1G). Again, slightly less infectious virus was released into the supernatant from tetherin-expressing cells at the earliest time of 14 h, although this difference was statistically insignificant. Moreover, infectious titers in the supernatants were equal by 24 h after infection and up to 48 hpi. Q-PCR detection of DNase-protected viral genomes confirmed this effect (Fig. 1H). Cell-associated virus titers at 14 hpi were very similar, and infected cell lysates showed similar levels of viral proteins, confirming that tetherin has no effect on viral DNA or protein synthesis (Fig. 1I and J). These data suggest that during high-multiplicity infection, tetherin is antagonized by HSV-1 infection. To test whether tetherin protein levels were impacted by HSV-1 infection, we measured them by immunoblotting at various time points after HSV-1 infection at an input multiplicity of 2 PFU/cell (Fig. 1K). We found that tetherin levels declined as viral protein VP5 increased. We assume that the different tetherin bands observed represent differently glycosylated forms (31). Importantly, all were reduced after infection. The loss of tetherin expression was somewhat specific, as β-actin and transferrin receptor protein levels were unaffected up to 16 h after infection and GAPDH levels were only slightly reduced (Fig. 1K). We next measured tetherin and GAPDH mRNA levels by quantitative RT-PCR in the same samples as Fig. 1K. We found that tetherin mRNA levels declined with a time course similar to that of protein levels, suggesting a role for the virus host shutoff (Vhs) function in which host mRNAs are degraded by the Vhs protein, encoded by the HSV-1 UL41 gene (Fig. 1L). GAPDH mRNA was also lost, although with less concomitant reduction in protein expression. We conclude that tetherin is partly antagonized through suppression of expression and that this likely accounts for the loss of restriction after high-multiplicity infection (Fig. 1G). These data are consistent with the recently reported antagonism of tetherin by the HSV-1 Vhs response (see the accompanying paper [32]) but do not rule out the possibility that tetherin might be degraded through additional mechanisms.

### Tetherin depletion with shRNA increases HSV-1 release.

**Fig 2:**
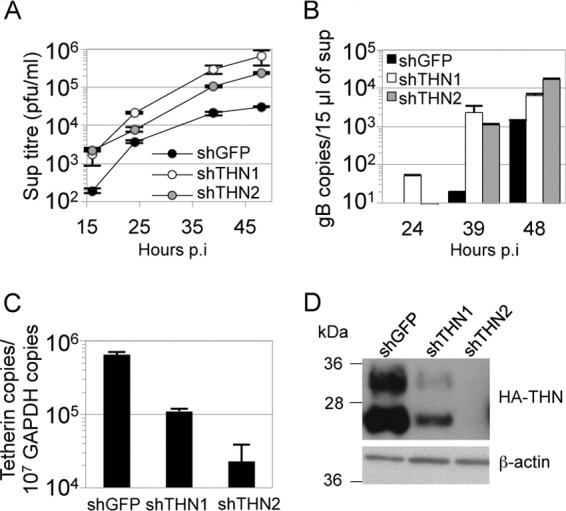
shRNA-mediated depletion of tetherin increases HSV-1 release. HeLa cells were transduced with HIV-1 vector encoding either of 2 tetherin-specific shRNAs or a shRNA targeting GFP (A to C). Cells were then infected with HSV-1 SC16 (0.1 PFU/cell). HSV-1 infectivity in the supernatants was measured by plaque assay (A) and that in DNase-protected genomes by Q-PCR (B). Tetherin mRNA depletion was assessed in HeLa by Q-PCR normalized to GAPDH (C). The increase in HSV-1 titers after tetherin depletion (A) was significant (2-way ANOVA; *P* < 0.05). Tetherin reduction was also assessed by immunoblotting HA tag (tetherin) or β-actin (after membrane stripping) in shRNA-transduced HA-tagged-tetherin-expressing HT1080 cells (D). Results are means and SD and are representative of 3 separate experiments.

To confirm that tetherin was responsible for the reduction in HSV-1 release seen in Fig. 1A, we depleted endogenous tetherin expression from HeLa cells and measured release of HSV-1 into the supernatant at various times after low-multiplicity infection (0.1 PFU/cell). HeLa cells are known to express amounts of tetherin that restrict HIV-1 strains lacking the tetherin antagonist Vpu (27). We used two tetherin-specific shRNAs and a control shRNA targeting GFP. Expression of either of the anti-tetherin shRNAs improved the release of virus, as indicated by an increase in infectious titer in supernatants particularly after 39 hpi, compared with the titer obtained from cells expressing shGFP (Fig. 2A). Q-PCR detection of DNase-protected genomes confirmed that less virus was released from cells expressing the GFP-specific shRNA (Fig. 2B). Quantitative RT-PCR showed that endogenous tetherin mRNA levels were reduced in HeLa cells expressing tetherin-specific shRNA compared to cells expressing the hairpin targeting GFP (Fig. 2C). The effect of shRNA expression was also confirmed by immunoblotting detecting the HA tag in extracts of HT1080 cells expressing HA-tagged tetherin (Fig. 2D) and transduced with shRNA-encoding lentivectors.

### Tetherin induces accumulation of HSV-1 particles at the cell surface.

**Fig 3:**
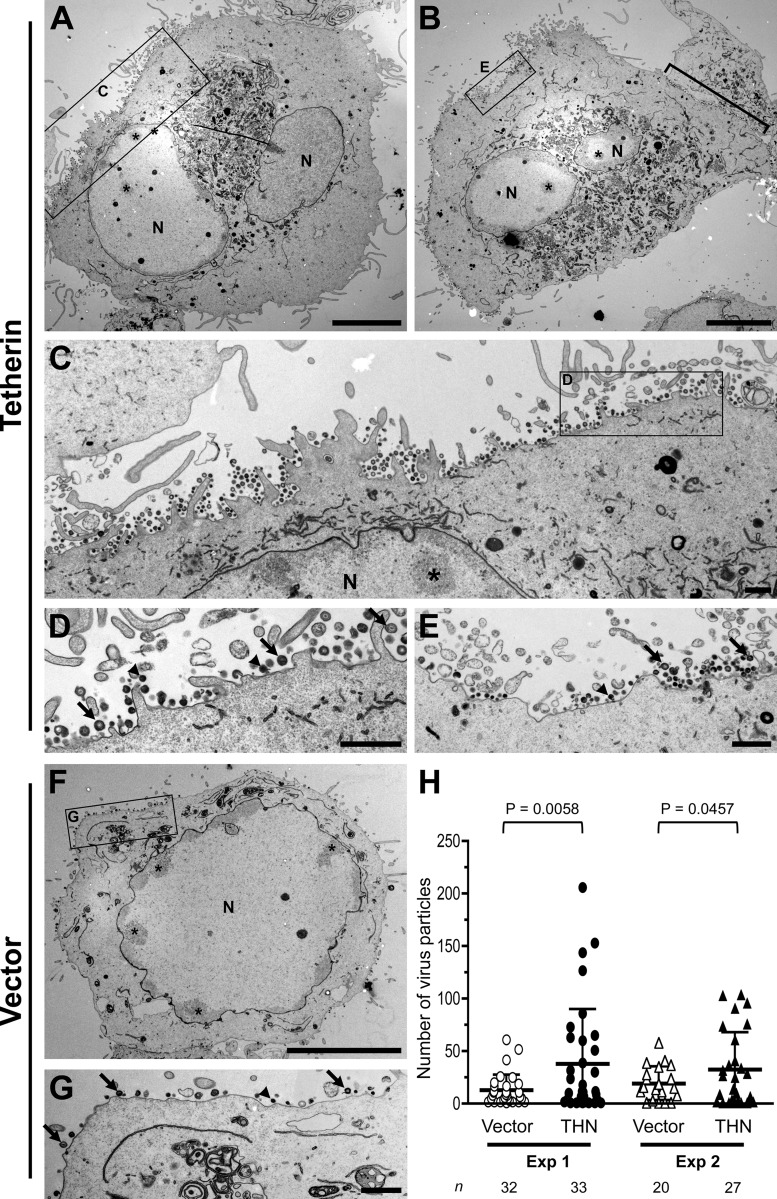
Tetherin retains HSV-1 particles at the cell surface. HT1080 cells expressing HA-tagged tetherin (A to E) or empty vector (F and G) and infected with HSV-1 K26GFP were examined by electron microscopy. The boxed areas in A, B, C, and F are shown at higher magnification in panels C, D, E, and G, as indicated. N, nucleus. The asterisks mark accumulations of viral nucleocapsids. The bracket marks a layer of viruses at the cell-cell interface in panel B. In panels D, E, and G, selected HSV-1 particles with typical morphology are indicated by the black arrows, while some of the more tangentially cut electron-dense viral profiles are marked with black arrowheads. Bars, 10 μm (A, B, and E) and 1 μm (C, D, E, and G). (H) Counts of cell surface virus particles from both experiments (Exp) are plotted. Open symbols, particle counts from cells expressing vector; filled symbols, counts from HA-tetherin expressing cells. The number of cells analyzed is indicated at the bottom. Values are means ± standard deviations; *P* values were determined using an unpaired *t* test with Welch's correction on cells having at least one viral particle on the cell surface. (Panels A to G show data from experiment 1.)

Having established that tetherin can restrict HSV-1 release, we sought to visualize restricted virus on the surfaces of tetherin-expressing cells. Thin-section electron microscopy revealed that in HSV-1-infected HT1080 cells that do not express tetherin, there were few virions associated with the cell surface. However, in infected cells overexpressing tetherin, there were areas of cell surface where many virions were associated with the plasma membrane (Fig. 3A to G). To quantify this effect, we counted cell surface-associated virions on control and tetherin-expressing cell profiles in a blinded manner. In two experiments, tetherin-expressing cells had significantly more cell surface virions per cell profile than control cells (Table 1; Fig. 3H). These observations are consistent with tetherin suppressing HSV-1 release from infected cells (Fig. 1).

**Table 1:** HSV-1 particle counting at the cell surface

Expt and cells^*a*^	No. of:			
	Cell profiles examined	Infected cells^*b*^	HSV-1 profiles at the cell surface	Cell surface HSV-1 particles per infected-cell profile
1				
Vector	60	32	419	13.09
THN	51	33	1,258	38.12
2				
Vector	62	20	389	19.45
THN	70	27	886	32.81

a THN, HT1080 cells expressing HA-tagged tetherin; Vector, HT1080 cells expressing empty vector.

b Cell profiles showing at least one viral particle at the cell surface.

### HSV-1 glycoprotein M can antagonize tetherin.

Viruses typically encode countermeasures to the repertoire of restriction factors expressed by their natural host. Numerous viral countermeasures to tetherin have been described (2, 4–8, 10, 11) which share one key mechanistic characteristic: they remove tetherin from the location of its antiviral activity. Importantly, glycoproteins from unrelated viruses, including lentiviruses (HIV-2 and SIVtan) (26, 29) and Ebola virus (33), have been shown to have anti-tetherin activity. With this in mind, we hypothesized that the HSV-1 envelope glycoprotein M (gM) may also act as a tetherin antagonist. This protein has been shown to relocalize membrane proteins (23, 34), and a deletion mutant replicates to reduced titers in a number of cell lines (23, 28, 35).

**Fig 4:**
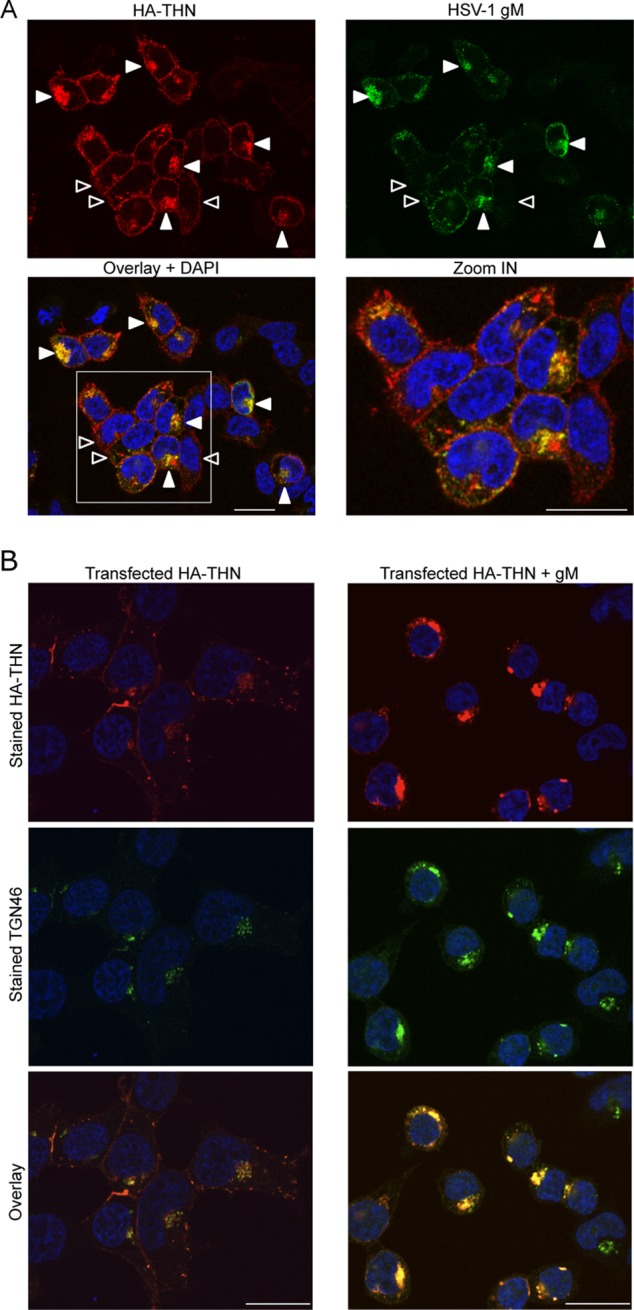
gM relocalizes tetherin to a TGN46-positive compartment. HEK293T cells expressing HA-tetherin alone or with HSV-1 gM were stained with anti-HA (red), anti-gM (A) or TGN46 (B) (green), and DAPI (blue; nuclei). (A) The last panel is an enlargement of the area marked on the Overlay + DAPI panel. Open arrowheads indicate tetherin predominantly localized to the plasma membrane in cells not expressing or expressing low levels of gM. Solid arrowheads (in cells staining brightly for gM) indicate tetherin localized to perinuclear regions and colocalized with gM. Images are representative of at least 2 separate experiments. Bars, 20 μm.

To investigate whether gM has a role in tetherin antagonism, we first used confocal immunofluorescence microscopy to investigate the effect of gM expression on tetherin localization. We cotransfected HEK293T cells with plasmids encoding HSV-1 gM and HA-tagged tetherin and examined their localization after 48 h (Fig. 4A). In cells that did not express gM, or expressed low levels of gM (Fig. 4A, open arrowheads), tetherin was predominantly localized to the plasma membrane, as described previously (2). However, in cells staining brightly for gM, a significant proportion of the tetherin was localized in the perinuclear region of the cell, where it overlapped with gM labeling (solid arrowheads). Further investigation indicated that the gM labeling also overlapped with labeling for the trans-Golgi network marker TGN46 (Fig. 4B), suggesting that tetherin-gM complexes are located in the TGN. Together, these data suggest that, like HIV-1 Vpu, gM can relocalize tetherin, consistent with its having a role in antagonizing the restriction of virus release.

**Fig 5:**
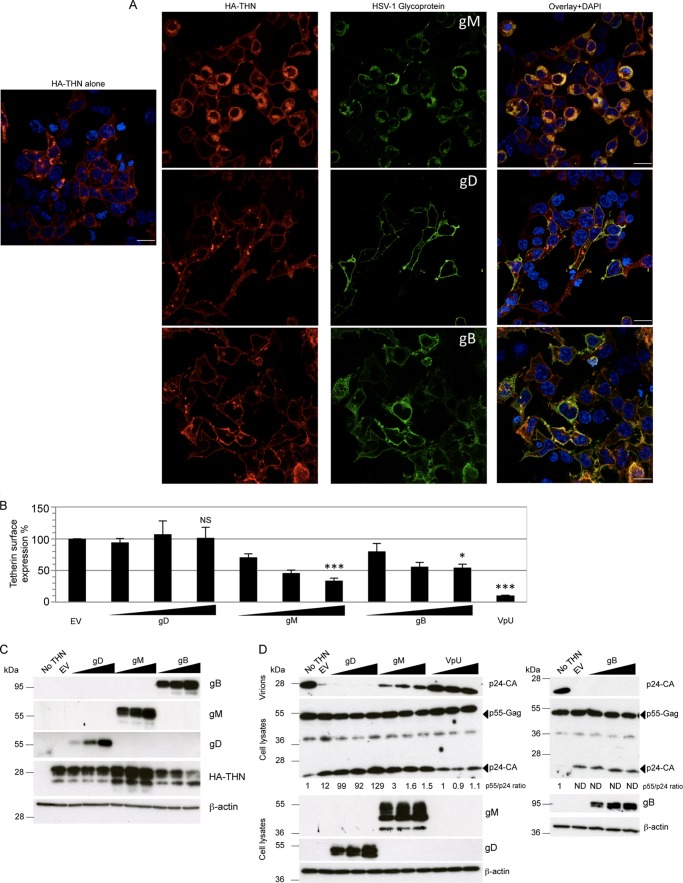
Ability of HSV-1 glycoproteins to remove tetherin from the cell surface and rescue HIV-1 from restriction. HEK293T cells expressing HA-tetherin alone or with HSV-1 gM, gD, or gB were stained with anti-HA (red) and anti-gM, anti-gD, or anti-gB (green), respectively, and DAPI (blue; nuclei). Images are representative of 2 separate experiments. Bars, 20 μm. (B) Flow-cytometric detection of cell surface tetherin on HEK293T cells expressing HA-tetherin alone (EV) or in combination with 3 different amounts (250, 500, and 1,000 ng) of gM, gD, or gB plasmids. Cells transfected with 1,000 ng of a Vpu plasmid were used as a control. Mean fluorescence intensities for tetherin are plotted as a percentage of the EV value. Data shown are means of 3 or 4 experiments ± SEM. Statistical significance was determined using a *t* test comparing mean fluorescence intensity from cells transfected with 1,000 ng of plasmids (*, *P* < 0.05; ***, *P* < 0.001; NS, not significant). (C) Cells transfected for the flow-cytometric assay (B) were used for immunoblotting gM, gD, gB, HA-tetherin, and β-actin, sequentially on the same membrane after stripping. (D) HEK293T cells were cotransfected with 3 HIV-1 vector plasmids and, in addition, an empty (no THN) or tetherin expression (EV) vector, or a tetherin expression vector plus 3 different amounts (as in panels B and C) of gM, gD, gB, or Vpu plasmids. p24 capsid (CA) in supernatants (virions) and p55 Gag expression in cell lysates, as well as glycoprotein expression, were analyzed 48 h later by immunoblotting, with β-actin used as a loading control after membrane stripping. Intracellular-p55/supernatant-p24 ratios are indicated below the p24-stained membranes and are relative to the ratio obtained with no tetherin. “ND” indicates that ratios could not be determined due to the absence of any p24 band. The image is representative of at least 3 experiments.

In order to assess the specificity of tetherin antagonism by gM, we compared the localization of tetherin after expression of gM and HSV-1 glycoproteins gD and gB. As before, we expressed the glycoproteins transiently, together with HA-tagged tetherin, in HEK293T cells and stained tetherin and each glycoprotein using specific antibodies. We found that, as before, gM caused a relocalization of tetherin from the plasma membrane to an internal compartment (Fig. 5A). On the other hand, gD had no such effect, and tetherin mostly localized to the cell surface, as in control cells. On expression of gB, we found that there was a less extensive relocalization of tetherin than after gM expression. We conclude that gD has no tetherin relocalization activity and that gB may have some activity but less than that of gM.

To examine tetherin antagonism by HSV-1 glycoproteins further, we used flow cytometry to quantify tetherin surface expression when tetherin was coexpressed with glycoproteins, again in HEK293T cells. Consistent with the immunofluorescence data, fluorescence-activated cell sorting analysis showed reduced levels of tetherin on the surfaces of gM-expressing cells compared to cells expressing empty vector (Fig. 5B). We used the HIV-1 tetherin antagonist Vpu as a positive control in these experiments. gD expression had no effect, whereas gB caused a small reduction of tetherin surface staining that was less than that caused by gM (Fig. 5B). All glycoproteins were expressed efficiently, as measured by immunoblotting (Fig. 5C), and there was a striking elevation in the amount of tetherin in the gM-expressing cells, consistent with the gM-driven accumulation in the TGN (Fig. 4B). The flow cytometry measurements (Fig. 5B) were therefore consistent with immunofluorescence staining of tetherin expression (Fig. 5A).

For a functional assessment of the antagonistic effect of HSV-1 glycoproteins on tetherin and to provide further mechanistic insight, we asked whether they could substitute for Vpu in an HIV-1 release assay (27). HIV-1 particles released into the supernatant were detected by immunoblotting for the HIV-1 Gag structural protein p24 at 48 h posttransfection. As expected, Vpu expression effectively antagonized tetherin restriction, and HIV-1 particles (p24-CA) were detected in the culture supernatant at levels equivalent to those obtained in the absence of tetherin (Fig. 5D). When we replaced Vpu with gM, we saw a comparable rescue of HIV-1 release (intracellular-p55/supernatant-p24 ratios are shown in Fig. 5D). These data demonstrate that gM can rescue HIV-1 release from tetherin restriction and are consistent with gM acting as an antagonist of the antiviral function of tetherin during HSV-1 infection. Importantly, neither gB nor gD was able to rescue HIV-1 release. Thus, while gB had a weak effect on tetherin localization, it was unable to functionally substitute for Vpu in an assay directly measuring functional antagonism of tetherin. These observations suggest that gM has specificity in antagonizing tetherin. Clearly, this model system depends on overexpression of Vpu and gM, likely in excess of the levels achieved during an infection; nevertheless, it allows a useful functional comparison between unrelated molecules and provides an independent demonstration that gM has anti-tetherin activity.

**Fig 6:**
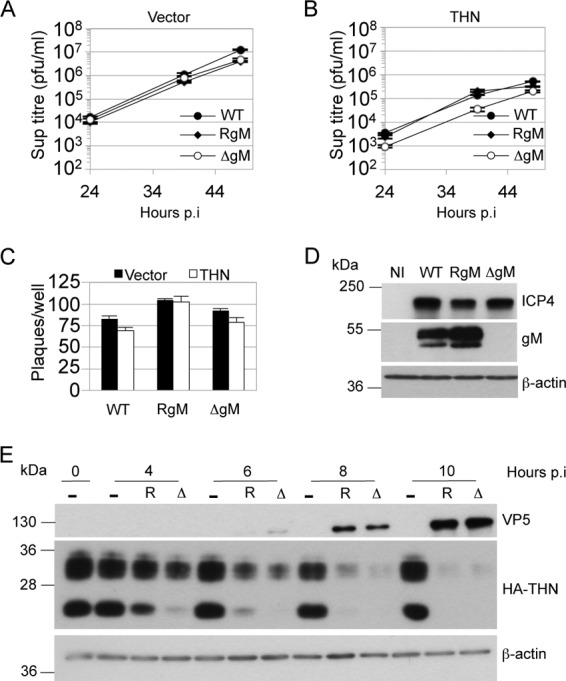
gM antagonizes tetherin during HSV-1 infection. Control (A) and HA-tagged-tetherin-expressing (B) HT1080 cells were infected with HSV-1 SC16 (WT), a gM deletion virus (ΔgM), or a revertant virus (RgM) at 0.01 PFU/cell, and supernatants harvested at the indicated times postinfection were titrated for HSV-1 infectivity by plaque assay. Two-way ANOVA of 3 separate tetherin expression experiments confirmed statistically significant differences attributable to time and the presence of gM (*P* < 0.01). (C) HSV-1 SC16 WT, ΔgM, and RgM were plaque assayed on cell lines used for panels A and B. Results are means and SEM and are from 2 independent experiments. (D) Immunoblot detection of ICP4, gM, and β-actin in lysates of HT1080 cells infected with the 3 viruses or noninfected (NI). (E) Immunoblot detection of VP5 and HA tag (tetherin), or β-actin after membrane stripping, in lysates of HA-tagged tetherin-expressing HT1080 cells infected with ΔgM (Δ) or RgM (R) virus at 2 PFU/cell or noninfected (−). Results are representative of 3 separate experiments.

We next asked whether the tetherin antagonism shown by gM facilitated release of HSV-1 from infected cells. We infected control and tetherin-expressing HT1080 cells with HSV-1 SC16 (WT), a gM deletion virus (ΔgM), or a revertant virus (RgM) using a low input multiplicity (0.01 PFU/cell). Titers of the three viruses released from control cells were indistinguishable (Fig. 6A). However, on cells overexpressing tetherin, while all three viruses were inhibited, the ΔgM virus was inhibited by a small but statistically significant degree over 48 h (2- to 3-fold) (Fig. 6B). We reasoned that, as in the experiments whose results are shown in Fig. 1, tetherin overexpression largely saturated HSV-1 tetherin antagonism, leading to inhibition of all three viruses. Slightly stronger restriction of the ΔgM virus was likely due to its reduced ability to antagonize tetherin. Importantly, there was no significant difference between the HT1080 lines in the number of plaques obtained for any of the viruses for a given dose (Fig. 6C). Absence of gM from ΔgM-infected cells was confirmed by immunoblotting for gM protein in infected-cell extracts (Fig. 6D). Finally, we infected HT1080 overexpressing HA-tagged tetherin with RgM or ΔgM viruses and analyzed cell lysates by Western blotting, detecting HA-tetherin, the HSV-1 capsid protein VP5, or β-actin as a loading control, at various time points postinfection (Fig. 6E). As early as 4 to 6 hpi, we observed a reduction of tetherin protein in infected cells compared to noninfected cells, as previously shown (Fig. 1K). Tetherin was almost completely lost by 10 hpi. gM expression was not responsible for loss of tetherin, as indicated by the observation that the ΔgM virus infection also led to tetherin loss. These data indicate that while HSV-1 gM can act as a tetherin antagonist, the virus has at least one other anti-tetherin activity responsible for loss of tetherin protein.

## DISCUSSION

Here we extend the repertoire of tetherin-restricted viruses to HSV-1 and the list of tetherin antagonists to include the HSV-1 glycoprotein gM. It is striking that glycoproteins are common as tetherin antagonists, with anti-tetherin activity being described for glycoproteins from the lentiviruses SIVtan and HIV-2 as well as the filovirus Ebola virus (4, 6, 10). Our data suggest a complex relationship between tetherin and HSV-1. Tetherin is clearly antagonized by HSV-1 in at least two independent ways, by gM-mediated relocalization and through a gM-independent suppression of tetherin mRNA (Fig. 1), likely through the Vhs response (32).

The modest effect of tetherin on HSV-1 replication in our experiments may be because HSV-1 is largely insensitive to tetherin restriction. However, we speculate that gM's role as a tetherin antagonist may not solely be to improve HSV-1 release. Rather, its presence within incoming virions might be important for suppressing tetherin innate signaling. Tetherin activates innate immune signaling cascades via NF-κB, inducing an innate immune response on engagement with virus (36). As a pattern recognition receptor, tetherin may be a particularly important target for early antagonism before Vhs takes effect. The ability of gM to rescue HIV-1 from tetherin restriction provides good evidence for functional tetherin antagonism by gM. Rescue of HIV-1 from tetherin restriction by gM appears to be more potent than rescue of HSV-1 from tetherin (compare Fig. 5, HIV-1, with Fig. 6, HSV-1). We assume that this is in part because, in the case of HIV-1, all tetherin antagonism is abrogated by deletion of Vpu, and thus tetherin restriction is maximal and entirely rescued by gM expression. However, in the case of HSV-1, in the absence of gM, tetherin is still antagonized by Vhs (32) and potentially other, as-yet-uncharacterized viral functions. Indeed, gB had a minor effect on tetherin localization measured by immunofluorescence staining and flow cytometry, although it had no effect in an HIV-1 tethering assay. gD had no measurable effect in any of our assays.

How gM achieves ligand specificity remains unknown, but we note that HIV-1 Vpu is also promiscuous, removing multiple proteins from the cell surface (37–40). It is unclear whether gM actively removes tetherin or whether, like HIV-1 Vpu, gM prevents tetherin from reaching the cell surface (41, 42). Regardless, the result is accumulation of tetherin, detected by immunoblotting (Fig. 5C) and the presence of tetherin in a compartment positive for TGN46 staining (Fig. 4B). Notably, HIV-2 Env also relocalizes tetherin to a TGN46 positive compartment without degrading it (6). Thus, it appears that, in common with other viral tetherin countermeasures, gM acts by removing tetherin from the site of virus budding, in this case TGN46-negative endocytic tubules (33). We envisage the interaction between tetherin and outgoing HSV-1 virions occurring in this compartment, and the relocalization of tetherin we observed by immunofluorescence (Fig. 4B) provides a plausible basis for the anti-tetherin activity of gM. We did not see any chains of tethered virions similar to those formed by tetherin-restricted lentiviruses (2). This may reflect spatial constraints imposed by budding into tubules. Indeed, we assume that newly budded tethered virions would not be readily apparent by electron microscopy because they would simply be restrained at the location in which they normally reside. However, following release, virions would remain tethered to the cell surface, as we observed (Fig. 3). This phenomenon may provide some explanation as to how tetherin restricts release of HSV-1 and not direct cell-to-cell spread, reflected by the lack of impact on plaque size. We assume that surface-tethered virions may be able to interact with target cell receptors to initiate infection despite being tethered to the infected cell membrane. Such a process has been described for the lentiviruses HIV-1 and FIV (29, 30).

Redundant herpesvirus-encoded tetherin antagonists are also found in KSHV (15, 16, 43). KSHV encodes the E3 ubiquitin ligase K5, which recruits tetherin to cause its degradation yet can still antagonize tetherin after K5 depletion with RNA interference (16). Thus, large viruses, such as herpesviruses, may encode several partially redundant tetherin antagonists that have subtly different roles in restriction factor antagonism. The complex relationship between herpesviruses and tetherin may also explain the puzzling result that while HSV-1 and KSHV are restricted by tetherin, this protein has been reported to act as a cofactor for HCMV replication (15–17). Tetherin expression improves HCMV infectivity a few-fold, perhaps related to its ability to stabilize lipid rafts, which play a key role in HCMV entry (44, 45). We did not observe an equivalent enhancement with HSV-1 (Fig. 1 and 2), and it remains unclear whether HCMV also encodes proteins that manipulate tetherin to prevent it from restricting the virus.

The increasing variety of viruses that are restricted by tetherin illustrates the power of a restriction factor that targets the fundamental processes of viral budding and release. The diversity of tetherin antagonists that viruses have evolved also emphasizes the importance of overcoming this system of host defense. It is likely that the study of the ongoing evolutionary conflict between viruses and tetherin, which is suggested by the Red Queen hypothesis (46), will lead to significant enhancements to our understanding of the cell biology of both viruses and their hosts and the relationships between them.
